# The Concordance between Mumps and Rubella Sero-Positivity among the Israeli Population in 2015

**DOI:** 10.3390/vaccines10070996

**Published:** 2022-06-22

**Authors:** Ravit Bassal, Tamy Shohat, Tal Levin, Rakefet Pando, Eilat Shinar, Doron Amichay, Mira Barak, Anat Ben-Dor, Adina Bar-Haim, Ella Mendelson, Dani Cohen, Lital Keinan-Boker, Victoria Indenbaum

**Affiliations:** 1 Israel Center for Disease Control, Ministry of Health, Gertner Institute, Chaim Sheba Medical Center, Tel Hashomer 52621, Israel; tamyshohat@gmail.com (T.S.); lital.keinan2@moh.gov.il (L.K.-B.); 2 Department of Epidemiology and Preventive Medicine, Sackler Faculty of Medicine, Tel-Aviv University, Tel-Aviv 69978, Israel; ella.mendelson@sheba.health.gov.il (E.M.); dancohen@tauex.tau.ac.il (D.C.); 3 Central Virology Laboratory, Public Health Services, Ministry of Health, Chaim Sheba Medical Center, Tel Hashomer 52621, Israel; tal.levin@sheba.gov.il (T.L.); rakefet.pando@sheba.health.gov.il (R.P.); viki.indenbaum@sheba.health.gov.il (V.I.); 4 Magen David Adom (MDA) National Blood Services, Tel Hashomer 52621, Israel; eilats@mda.org.il; 5 Soroka University Medical Center, Clalit Health Services, Beer Sheva 84101, Israel; dorona@clalit.org.il; 6 Haifa and Western Galilee Laboratories, Clalit Health Services, Nesher 20300, Israel; mirabarak@zefat.ac.il; 7 Zefat Academic College, Safed 13206, Israel; 8 Schneider Children Medical Center, Clalit Health Services, Petah-Tiqwa 49504, Israel; anatbe4@clalit.org.il; 9 Mayanei HaYeshuhai Medical Center, Bnei Brak 51544, Israel; dinab@asaf.health.gov.il; 10 School of Public Health, University of Haifa, Haifa 3498838, Israel

**Keywords:** mumps, rubella, sero-prevalence, virus, vaccine

## Abstract

Mumps and rubella are vaccine-preventable viral diseases through the measles-mumps-rubella-varicella (MMRV) vaccine, administered at 12 months and again at 6 years. We assessed the sero-prevalence of mumps and rubella, identified factors associated with sero-negativity, and evaluated concordance between mumps and rubella sero-positivity. A national cross-sectional sero-survey was conducted on samples collected in 2015 by the Israel National Sera Bank. Samples were tested for mumps and rubella IgG antibodies using an enzyme-linked immunosorbent assay. Of 3131 samples tested for mumps IgG, 84.8% (95%CI: 83.5–86.0%) were sero-positive. Sero-negativity for mumps was significantly associated with age (high odds ratios observed in infants younger than 4 years and 20–29 years old subjects). Of 3169 samples tested for rubella IgG antibodies, 95.2% (95%CI: 94.4–95.9%) were sero-positive. Rubella sero-negativity was significantly associated with age (high odds ratios observed in children younger than 4 years old and adults older than 30 years), males, Jews, and others. Concordant sero-positivity for both mumps and rubella viruses was observed in 83.9% of the tested samples. The Israeli population was sufficiently protected against rubella but not against mumps. Since both components are administered in the MMRV vaccine simultaneously, the mumps component has a lower uptake than rubella and quicker waning.

## 1. Introduction

Mumps and rubella are vaccine preventable viral diseases. In Israel, mumps vaccination was provided to infants aged 15 months from 1984 until June 1985, was suspended from July 1985 to 1988, and was reintroduced at the end of 1988 for the same age group. Since 1988, the mumps vaccine has been administered in combination with measles and rubella (MMR) and since 2007, in combination with measles, rubella and varicella (MMRV) in two doses, at the age of 12 months and 6 years [1]. The incidence rate of mumps in Israel between 1977 and 1988 ranged between 79.8 and 157.9 per 100,000 and has since decreased [2]. In 2021, the incidence rate of mumps in Israel was 0.3 per 100,000. Though mumps incidence rates declined dramatically, outbreaks still occur, as reported in Israel in 2009, 2010, and 2011, with incidence rates of 10.4, 64.6, and 3.9 per 100,000, respectively [1,2].

The incidence rate of rubella disease in 1972 was 547.7 per 100,000, but following the introduction of one vaccine dose in 1973 for girls in the 6th grade, the incidence rate declined to less than 31.3 per 100,000 [1,2]. Yet, rubella outbreaks were still documented in 1978–1979, 1983–1984, 1987–1988, and 1992, with incidence rates as high as 959.6 per 100,000 in 1979 [1,2]. In 1988, vaccination was extended to boys, and in 1994 was administered as a two-dose vaccine for both boys and girls at 12 months of age and in 6th grade as part of the MMR vaccine [1]. The two-dose vaccine schedule led to a dramatic decline in the incidence rate of rubella cases in Israel [1,2].

In this study, we aimed to assess the sero-prevalence of mumps and rubella IgG antibodies, to identify factors associated with sero-negativity, and to evaluate the concordance between mumps and rubella sero-positivity among the Israeli population in 2015.

## 2. Methods

*Sampling:* 3169 serum samples were obtained from the Israel National Sera Bank (INSB), which was established in 1997 by the Israel Center for Disease Control. The samples included in the study were collected in 2015 from the following laboratories: 1. Haifa and Western Galilee Clalit Health Maintenance Organization (HMO) located in northern Israel, 2. The National Bank Service, a division of Magen David Adom (MDA) located in central Israel (though collects samples from all over Israel), 3. Schneider Children Medical Center located in central Israel, 4. Mayanei HaYeshuhai Medical Center located in central Israel, and 5. Soroka Clalit HMO (non-hospitalized patients) located in southern Israel. For each sample, the following data were collected: age group, gender, birth country, and population group [Jews and others (others are Non-Arab Christians and citizens with no defined ethnicity) vs. Arabs]. In addition, we collected data on the district of residence and socio-economic status (SES) (allocated using the classification of the Israeli Central Bureau of Statistic set for each address, categorized from low (1–5) to high (6–10) [3]). The specific methods, representativeness, and the challenges of the INSB sample collection were described previously [4].

*Ethics*: The INSB sample collection was approved by the legal department of the Israeli Ministry of Health and is completely anonymous.

*Laboratory Methods:* Serological tests for the detection of mumps and rubella-specific IgG antibodies were performed at the Central Virology Laboratory of the Israeli Ministry of Health, using the enzyme-linked immunosorbent assay (ELISA) commercial kits for mumps [Enzygnost^®^ Anti-Parotitis-Virus/IgG (Marburg, Germany), kit sensitivity: 95.4%; specificity: 93.7%] and rubella [Enzygnost^®^ Anti-Rubella Virus/IgG (Marburg, Germany), kit sensitivity: 100.0%; specificity: 98.5%]. The samples were classified as follows: optical density (OD) > 0.2 was considered positive, 0.1–0.2 equivocal, and <0.1 negative. For both mumps and rubella, equivocal samples were classified as positive. All samples (3169 samples) were tested for rubella and 3131 samples were tested for mumps due to lack of biological material.

*Data Analysis:* Sero-prevalence rates were calculated using proportions and 95% confidence intervals (CI). Logistic regression analyses were performed to evaluate the variables associated with sero-negativity. Age-adjusted odds ratios (OR) and 95% CI were calculated. Due to sparse data, we used the penalized logistic regression (PLR) method [5]. The goodness-of-fit of the models was assessed using c-statistic. Weighted kappa (ĸ) was calculated for the concordance between the sero-prevalence of mumps and rubella. SAS Enterprise Guide software package (version 7.12, SAS Institute Inc., Cary, NC, USA) was used for statistical analyses.

## 3. Results

Mumps: Of the 3131 samples tested, 2655 (84.8%; 95%CI: 83.5–86.0%) were positive for mumps IgG and 476 (15.2%; 95%CI: 14.0–16.5%) were negative. Demographic characteristics and sero-positivity rates are presented in Table 1.

Sero-positivity rates differed significantly by age (*p*-value < 0.0001) and districts of residence (*p*-value = 0.0005). Sero-positivity rate for mumps IgG was 40.0% among infants younger than 6 months and 3.8% among infants aged 6–11 months. Among those aged 1–4 years, sero-positivity was 79.5%, which increased to 88.0% in the 5–9 age range, and to 89.4% in the range of 10–14 years. The rates decreased to 82.8% in the 20–29 range, increased to 88.5% in the 30–34 age group, and 90.7% in the 35–44 range. In the age groups 45–54, 55–64, and 65+ years, sero-positivity rates were 94.0%, 92.0%, and 92.0%, respectively. Sero-positivity rates among residents of Jerusalem and the North districts were 88.7%, 88.4% in Haifa, 83.1% in the South, 83.0% in Judea and Samaria, 81.5% in Tel-Aviv, and 81.2% in the Central district. Table 2 represents the association between the demographic characteristics and sero-negativity for mumps.

In the multivariable analysis, sero-negativity for mumps was significantly associated with age group and district of residence. A significant high odds ratio was observed in infants younger than 6 months (OR = 9.95; 95% CI: 5.12–19.34), infants aged 6–11 months (OR = 173.46; 95% CI: 41.02–733.45), 1–4 years (OR = 1.83; 95% CI: 1.26–2.66), and 20–29 years (OR = 1.51; 95% CI: 1.03–2.22), compared with the 5–9 years age group. A significant low odds ratio for mumps sero-negativity was observed for those aged 45–54 years (OR = 0.46; 95% CI: 0.24–0.88), compared with the 5–9 years age group. Significant low odds ratios for sero-negativity were also observed for residents of the North (OR = 0.64; 95% CI: 0.46–0.87) and Haifa (OR = 0.62; 95% CI: 0.41–0.93) districts, compared with the Central district. The c-statistic of the multivariable analysis, including age group and district, was 0.676.

Rubella*:* Of the 3169 samples tested, 3016 (95.2%; 95% CI: 94.4–95.9%) were positive and 153 (4.8%; 95% CI: 4.1–5.6%) were negative for rubella. The demographic characteristics and sero-positivity rates for rubella are presented in Table 3.

Significant differences in sero-positivity rates were observed for age group (*p*-value < 0.0001), gender (*p*-value = 0.0006), population group (*p*-value = 0.0448), district of residence (*p*-value = 0.0001), and SES (*p*-value = 0.0397). Sero-positivity rates among infants younger than 6 months was 80.4%, decreased to 13.0% among infants aged 6–11 months, and increased to rates higher than 92.0% in the following age groups. Females had higher sero-positivity rates than males (96.5% vs. 93.9%) and Arabs had higher sero-positivity rates than Jews and Others (96.0% vs. 94.5%). Sero-positivity rates among residents of the Jerusalem district was 98.6%, Judea and Samaria 97.4%, North 97.2%, South 95.6%, Haifa 95.2%, Central 92.4%, and Tel Aviv 92.0%. The sero-positivity rate was significantly higher among those who were of high SES compared with low SES (95.8% vs. 94.1%).

**Figure 1:**
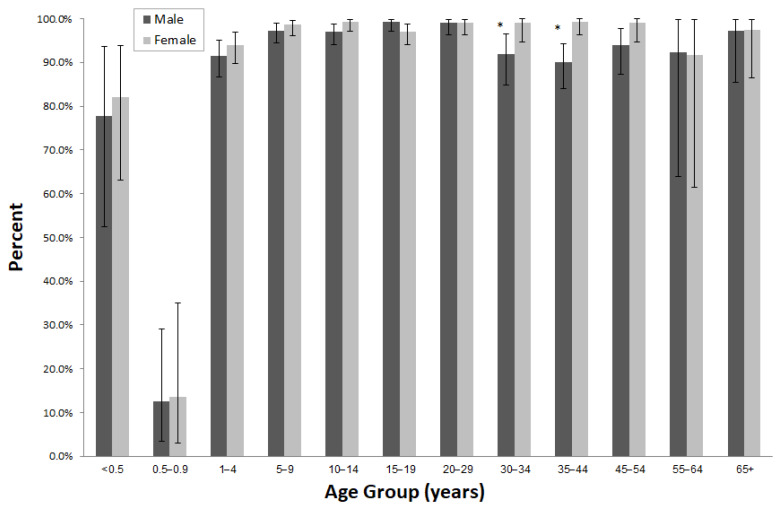
Sero-positivity for rubella by age group and gender. * p-value < 0.05.

Figure 1 demonstrates the differences between sero-positivity for rubella between males and females by age groups.

Sero-positivity rates for rubella were significantly higher in females than males in the 30–34 and 35–44 age groups.

Univariable and multivariable analyses for sero-negativity for rubella IgG by demographic characteristics are presented in Table 4.

Due to an association between district of residence and SES, only the district of residence was included in the best multivariable analysis (c-statistic = 0.810). Compared with the age group of 5–9 years, significantly higher odds for being sero-negative for rubella were observed almost in all other age groups: infants younger than 6 months (OR = 44.56; 95% CI: 43.92–45.21), infants aged 6–11 months (OR = 17.57; 95% CI: 10.06–25.08), 1–4 years (OR = 3.01; 95% CI: 2.98–3.04), 30–34 years (OR = 1.77; 95% CI: 1.74–1.80), 35–44 years (OR = 2.06; 95% C: 2.04–2.09), 45–54 (OR = 1.44; 95% CI: 1.41–1.46), 55–64 (OR = 5.06; 95% CI: 4.92–5.20), and 65+ (OR = 1.32; 95% CI: 1.28–1.36) years. Significant high odds ratios for rubella sero-negativity were also observed for males (OR = 1.56; 95% CI: 1.55–1.57) as well as for Jews and others (OR = 1.10; 95% CI: 1.10–1.11).

The concordance between mumps and rubella performed on 3131 samples is presented in Table 5 (ĸ = 0.3384, 95% CI: 0.2905–0.3863).

**Figure 2:**
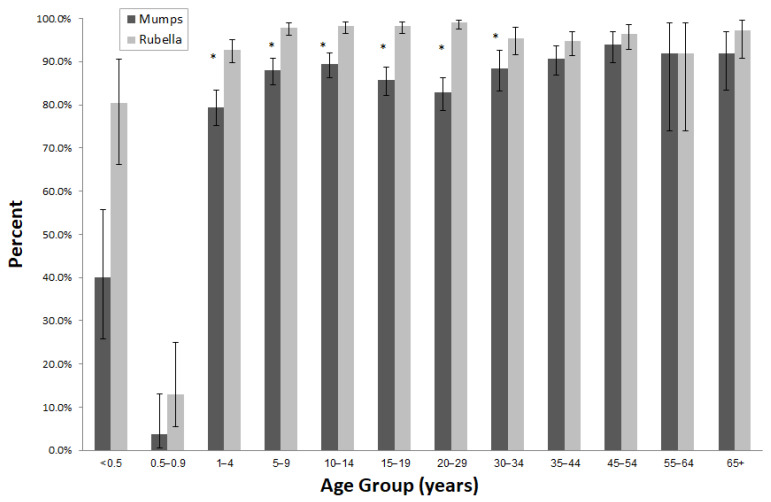
Sero-positivity against mumps and rubella by age group. * p-value < 0.05.

Overall, 83.9% were sero-positive for both mumps and rubella. The differences in sero-positivity rates for mumps and rubella by age group are presented in Figure 2.

Compared with sero-positivity for mumps, significantly higher rubella sero-positivity rates were observed in infants younger than 6 months, 1–4, 5–9, 10–14, 15–19, 20–29, and 30–34 years (Figure 2).

## 4. Discussion

In the current study, we have demonstrated relatively low sero-positivity rates for mumps (84.8%) and high sero-positivity rates for rubella (95.2%) in the Israeli population in 2015. The sero-positivity rate observed for mumps was lower than the rates observed in Colombia, among those 6–64 years old (91.6%) [6], in Catalonia, Spain, among those ≥15 years (91.1%) [7], in Madrid, Spain among those 19–39 years old (88.3%) [8], and in the United States among those 6–49 years old (87.6%) [9]. However, our reported mumps sero-positivity rates were higher than the rates reported in China among all age groups (82.0%) [10], Thailand among those 0–59 years old (82.0%) [11], in Taiwan among all age groups (71.0%) [12], and in the Czech Republic among adults ≥18 years (55.3%) [13]. The herd immunity threshold for mumps, according to Anderson and May, is 90–92% [14], higher than the sero-positivity rates observed in our study. However, it has been shown that preventing outbreaks and controlling mumps requires several elements, including high vaccine coverage (>95%) with the MMR/MMRV vaccine, with 4–8 years between doses [15]. The MMRV vaccine coverage in Israel in 2015 at the age of 24 months was 97% [16]. As explained, the first dose is administered at 12 months with a 5 year gap between the first and second doses (6 years), but the sero-positivity rates found in our study were relatively low and failed to reach these thresholds.

We have shown that age was a significant predictor for the serological status of mumps, as was previously reported by others [10,17]. In the first 6 months of life, sero-positivity rates for mumps were only 40.0%, pursuant to the transmission of antibodies from the mother to her fetus. The rate was 3.8% between the ages of 6 and 11 months, before administration of the first vaccine dose, and increased to 79.5% in the 1–4 year age group, probably as a result of vaccination at 12 months old, which is partial (one dose only). The low sero-positivity rates in the 20–29 age group reported in our study are consistent with the low sero-positivity rates observed in a sero-survey performed previously in Israel on samples collected between 1997 and 1998 [18]. However, significant high odds ratios for mumps sero-negativity were observed among the 20–29 age group, including those who were born between July 1985 and the end of 1988, when vaccination against mumps was suspended. The decline in mumps sero-positivity rates between the 10–14 age group and the 20–29 age group, who were vaccinated with two doses, can be explained by waning immunity, as was also reported by others [10,12,13,19]. The waning immunity may affect adolescents and young adults who are highly susceptible to mumps outbreaks, and indeed, these age groups were affected mostly in the 2009–2011 outbreak that occurred in Israel [20]. Another possible explanation for the low sero-positivity rates of mumps observed among those who were vaccinated was the low uptake of the mumps component in the MMR/MMRV vaccine, as was previously reported [9]. The addition of a third MMR/MMRV dose administered during outbreaks to the exposed population may decrease the risk for infection, especially for high-risk populations [21]. Though a third dose has a demonstrated limited value for routine use in a vaccinated population [13].

For rubella, we have shown that in Israel in 2015, 95.2% of the samples were sero-positive, a rate as high as in Catalonia, Spain among those ≥15 years (98.1%) [7], the United States (95.3%) [9], the Netherlands (95%) [22], Luxembourg (94.4%) [17], Madrid, Spain (94.4%) [8], and Australia (92.1%) [23]. The sero-positivity rate for rubella reported in our study was higher than the estimated herd immunity threshold of 83–85% required to prevent outbreaks of rubella [24]. The data are consistent with the low incidence rate of rubella infections observed in Israel.

In the first 6 months of life, sero-positivity rate for rubella was 80.4%, due to maternal antibodies. Between the ages of 6 and 11 months, the sero-positivity rate was much lower, 13.0%. Following the administration of the first rubella dose at 12 months, the sero-positivity rate in the 1–4 age group increased to 92.8%, but the odds for sero-negativity were still significantly high. High odds for rubella susceptibility in these age groups was also demonstrated in the Netherlands [22] and Germany [25]. Possible explanation for the significant high odds of sero-negativity in the 1–4 age group may be a delay in administration of the first vaccine dose.

We have shown that the sero-positivity of rubella antibodies in males was significantly lower than that of females in Israel, as was also reported by others [9,10,25,26], suggesting that males are more susceptible than females to rubella infections. The explanation for this finding may be the tendency for women to monitor their rubella vaccine status and close vaccination gaps at the fertility period, because of the concern of congenital rubella syndrome. In addition, the initial immunization program between 1973 and 1988 targeted only females, and males were not vaccinated. This is substantially reflected in the difference in the sero-positivity rates of males and females in the 35 –44 age group.

The relatively low concordance in sero-positivity rates between mumps and rubella (83.9%) and the low sero-positivity rates of mumps compared with rubella induced by the MMR/MMRV vaccine, administered simultaneously in the same vaccine dose may be due to the low uptake of the mumps component in the vaccine and to waning immunity among adolescents.

The main limitation of our study is that vaccination history was not known due to sample anonymity and was assumed based on age and on the national vaccination schedule. Though the samples were collected in 2015, we should not expect any changes in the sero-positivity rates in Israel in 2022, as the vaccination schedule has not changed. Apart from small and local incidences, no major mumps or rubella outbreaks have been observed since. In addition, our study results used sero-positivity rates, which do not necessarily reflect effective immunity and protection against infection.

The advantages of our study are as follows: the number of samples tested was high and was satisfying in representing the composition of the Israeli population. We were also able to examine the concordance of the mumps and rubella components within the MMR/MMRV vaccine, as antibodies were measured in the same samples.

In conclusion, this study has demonstrated that the Israeli population was sufficiently protected against rubella but not against mumps in 2015. Both components are administered simultaneously through the MMR/MMRV vaccine, thus the rubella component has better uptake than mumps.

## Figures and Tables

**Table 1:** Sero-positivity for mumps IgG antibodies by demographic characteristics in Israel, 2015 (*n* = 3131).

		Tested		Sero-Positive			
		N	%	N	%	95% CI ^£^	*p*-Value
**Age group (years)**	**<0.5**	45	1.4	18	40.0	25.7–55.7	<0.0001
	**0.5–0.9**	53	1.7	2	3.8	0.5–13.0	
	**1–4**	395	12.6	314	79.5	75.2–83.4	
	**5–9**	467	14.9	411	88.0	84.7–90.8	
	**10–14**	489	15.6	437	89.4	86.3–92.0	
	**15–19**	482	15.4	413	85.7	82.2–88.7	
	**20–29**	400	12.8	331	82.8	78.7–86.3	
	**30–34**	200	6.4	177	88.5	83.2–92.6	
	**35–44**	300	9.6	272	90.7	86.8–93.7	
	**45–54**	200	6.4	188	94.0	89.8–96.9	
	**55–64**	25	0.8	23	92.0	74.0–99.0	
	**65+**	75	2.4	69	92.0	83.4–97.0	
**Sex**	**Male**	1581	50.5	1322	83.6	81.7–85.4	0.0634
	**Female**	1550	49.5	1333	86.0	84.2–87.7	
**Birth country**	**Israel**	2957	94.5	2507	84.8	83.4–86.1	0.9463
	**Others**	173	5.5	147	85.0	78.8–89.9	
**Population group**	**Jews and Others**	1750	55.9	1494	85.4	83.6–87.0	0.3137
	**Arabs**	1381	44.1	1161	84.1	82.0–86.0	
**District**	**Jerusalem**	71	2.3	63	88.7	79.0–95.0	0.0005
	**North**	820	26.2	727	88.7	86.3–90.8	
	**Haifa**	354	11.3	313	88.4	84.6–91.6	
	**Central**	576	18.4	468	81.2	77.8–84.4	
	**Tel-Aviv**	292	9.3	238	81.5	76.6–85.8	
	**South**	906	28.9	753	83.1	80.5–85.5	
	**Judea and Samaria**	112	3.6	93	83.0	74.8–89.5	
**Socioeconomic status (SES)**	**Low**	2014	64.3	1689	83.9	82.2–85.4	0.0506
	**High**	1117	35.7	966	86.5	84.3–88.4	

^£^ CI—Confidence interval.

**Table 2:** The association between demographic characteristics and sero-negativity for mumps in Israel, 2015.

		Sero-negativity-Univariable			Sero-negativity-Multivariable		
		OR ^€^	95% CI ^£^	*p*-Value	OR ^€^	95% CI ^£^	*p*-Value
**Age group (years)**	**<0.5**	11.01	5.70–21.27	<0.0001	9.95	5.12–19.34	<0.0001
	**0.5–0.9**	187.15	44.34–790.00	<0.0001	173.46	41.02–733.45	<0.0001
	**1–4**	1.89	1.31–2.74	0.0007	1.83	1.26–2.66	0.0015
	**5–9**	Ref.					
	**10–14**	0.87	0.58–1.30	0.5077	0.86	0.58–1.29	0.4752
	**15–19**	1.23	0.84–1.79	0.2905	1.21	0.83–1.77	0.3220
	**20–29**	1.53	1.04–2.24	0.0287	1.51	1.03–2.22	0.0351
	**30–34**	0.95	0.57–1.60	0.8572	0.94	0.56–1.58	0.8137
	**35–44**	0.76	0.47–1.22	0.2511	0.73	0.45–1.18	0.1965
	**45–54**	0.47	0.24–0.90	0.0216	0.46	0.24–0.88	0.0186
	**55–64**	0.64	0.15–2.78	0.5498	0.66	0.15–2.86	0.5746
	**65+**	0.64	0.26–1.54	0.3170	0.62	0.26–1.50	0.2904
**Sex**	**Male**	1.20	0.99–1.46	0.0637			
	**Female**	Ref.					
**Birth country**	**Israel**	1.02	0.66–1.56	0.9465			
	**Others**	Ref.					
**Population group**	**Jews and Others**	0.90	0.74–1.10	0.3139			
	**Arabs**	Ref.					
**District**	**Jerusalem**	0.55	0.26–1.18	0.1258	0.64	0.30–1.39	0.2584
	**North**	0.55	0.41–0.75	0.0001	0.64	0.46–0.87	0.0053
	**Haifa**	0.57	0.38–0.84	0.0041	0.62	0.41–0.93	0.0209
	**Central**	Ref.					
	**Tel-Aviv**	0.98	0.68–1.41	0.9269	0.90	0.60–1.35	0.6119
	**South**	0.88	0.67–1.16	0.3590	0.89	0.66–1.20	0.4463
	**Judea and Samaria**	0.88	0.52–1.51	0.6560	0.92	0.52–1.62	0.7729
**Socioeconomic status (SES)**	**Low**	1.23	1.00–1.52	0.0509			
	**High**	Ref.					

^€^ OR—Odds Ratio. ^£^ CI—Confidence interval.

**Table 3:** Sero-positivity for rubella IgG antibodies by demographic characteristics in Israel, 2015 (*n* = 3169).

		Tested		Sero-Positive			
		N	%	N	%	95% CI ^£^	*p*-Value
**Age group (years)**	**<0.5**	46	1.4	37	80.4	66.1–90.6	<0.0001
	**0.5–0.9**	54	1.7	7	13.0	5.4–24.9	
	**1–4**	400	12.6	371	92.8	89.8–95.1	
	**5–9**	480	15.2	470	97.9	96.2–99.0	
	**10–14**	500	15.8	491	98.2	96.6–99.2	
	**15–19**	489	15.4	480	98.2	96.5–99.2	
	**20–29**	400	12.6	396	99.0	97.5–99.7	
	**30–34**	200	6.3	191	95.5	91.6–97.9	
	**35–44**	300	9.5	284	94.7	91.5–96.9	
	**45–54**	200	6.3	193	96.5	92.9–98.6	
	**55–64**	25	0.8	23	92.0	74.0–99.0	
	**65+**	75	2.4	73	97.3	90.7–99.7	
**Sex**	**Male**	1600	50.5	1502	93.9	92.6–95.0	0.0006
	**Female**	1569	49.5	1514	96.5	95.5–97.4	
**Birth country**	**Israel**	2995	94.5	2852	95.2	94.4–96.0	0.5486
	**Others**	173	5.5	163	94.2	89.6–97.2	
**Population group**	**Jews and Others**	1781	56.2	1683	94.5	93.3–95.5	0.0448
	**Arabs**	1388	43.8	1333	96.0	94.9–97.0	
**District**	**Jerusalem**	72	2.3	71	98.6	92.5–100.00	0.0001
	**North**	823	26.0	800	97.2	95.8–98.2	
	**Haifa**	357	11.3	340	95.2	92.5–97.2	
	**Central**	593	18.7	548	92.4	90.0–94.4	
	**Tel-Aviv**	302	9.5	278	92.0	88.4–94.8	
	**South**	908	28.6	868	95.6	94.0–96.8	
	**Judea and Samaria**	114	3.6	111	97.4	92.5–99.4	
**Socioeconomic status (SES)**	**Low**	2028	64.0	1942	95.8	94.8–96.6	0.0397
	**High**	1141	36.0	1074	94.1	92.6–95.4	

^£^ CI—Confidence interval.

**Table 4:** The association between demographic characteristics and sero-negativity for rubella in Israel, 2015, univariable and multivariable analyses.

		Sero-Negativity—Univariable			Sero-Negativity—Multivariable		
		OR ^€^	95% CI ^£^	*p*-Value	OR ^€^	95% CI ^£^	*p*-Value
**Age group (years)**	**<0.5**	47.84	47.16–48.52	<0.0001	44.56	43.92–45.21	<0.0001
	**0.5–0.9**	18.05	8.85–27.25	0.0001	17.57	10.06–25.08	<0.0001
	**1–4**	2.98	2.95–3.01	<0.0001	3.01	2.98–3.04	<0.0001
	**5–9**	Ref.					
	**10–14**	0.94	0.93–0.95	<0.0001	0.97	0.96–0.98	<0.0001
	**15–19**	0.95	0.94–0.96	<0.0001	0.99	0.98–1.00	0.0557
	**20–29**	0.80	0.79–0.81	<0.0001	0.85	0.84–0.86	<0.0001
	**30–34**	1.71	1.68–1.74	<0.0001	1.77	1.74–1.80	<0.0001
	**35–44**	2.01	1.98–2.03	<0.0001	2.06	2.04–2.09	<0.0001
	**45–54**	1.38	1.36–1.41	<0.0001	1.44	1.41–1.46	<0.0001
	**55–64**	4.98	4.84–5.12	<0.0001	5.06	4.92–5.20	<0.0001
	**65+**	1.26	1.22–1.30	<0.0001	1.32	1.28–1.36	<0.0001
**Sex**	**Male**	1.80	1.28–2.52	0.0007	1.56	1.55–1.57	<0.0001
	**Female**	Ref.					
**Birth country**	**Israel**	Ref.					
	**Others**	1.22	0.63–2.37	0.5489			
**Population group**	**Jews and Others**	1.41	1.01–1.98	0.0458	1.10	1.10–1.11	<0.0001
	**Arabs**	Ref.					
**District**	**Jerusalem**	0.17	0.02–1.26	0.0836	0.57	0.55–0.59	<0.0001
	**North**	0.35	0.21–0.58	<0.0001	0.58	0.58–0.59	<0.0001
	**Haifa**	0.61	0.34–1.08	0.0903	0.77	0.76–0.78	<0.0001
	**Central**	Ref.					
	**Tel-Aviv**	1.05	0.63–1.76	0.8492	0.84	0.83–0.85	<0.0001
	**South**	0.56	0.36–0.87	0.0099	0.60	0.59–0.60	<0.0001
	**Judea and Samaria**	0.33	0.10–1.08	0.0664	0.55	0.54–0.56	<0.0001
**Socioeconomic status (SES)**	**Low**	Ref.					
	**High**	1.41	1.02–1.96	0.0405			

^€^ OR—Odds Ratio. ^£^ CI—Confidence interval.

**Table 5:** Concordance between the sero-prevalence of mumps and rubella.

		Rubella Sero-prevalence		
**Mumps sero-prevalence**		**Negative** **N (%)**	**Positive** **N (%)**	**Total** **N (%)**
	**Negative N (%)**	121 (3.9)	355 (11.3)	476 (15.2)
	**Positive N (%)**	29 (0.9)	2626 (83.9)	2655 (84.8)
	**Total N (%)**	150 (4.8)	2981 (95.2)	3131 (100.0)

## Data Availability

The data presented in this study are available on request from the corresponding author. The data are not publicly available due to privacy restrictions. All data that were analyzed during this study are included in this published article.
